# Retroperitoneal bronchogenic cyst mimicking an adrenal cyst: case report

**DOI:** 10.1093/bjrcr/uaad001

**Published:** 2023-12-13

**Authors:** Hajra Idrees, Raza Zarrar, Ceylan A Taslicay, Khaled M Elsayes

**Affiliations:** Department of Musculoskeletal Radiology, University of Texas MD Anderson Cancer Center, Houston 77030, United States; Internal Medicine Department, Baptist Hospitals of Southeast Texas, Beaumont 77701, United States; Department of Diagnostic Radiology, University of Texas MD Anderson Cancer Center, Houston 77030, United States; Department of Abdominal Radiology, University of Texas MD Anderson Cancer Center, Houston 77030, United States

**Keywords:** retroperitoneum, bronchogenic cyst, Computed Tomography, magnetic resonance imaging

## Abstract

Bronchogenic cysts are rare lesions that form during early embryogenesis and are commonly located in the mediastinum. Retroperitoneal bronchogenic cysts (RBs) are exceptionally rare, with only a handful of cases reported in the modern literature. Here, we report an RB found incidentally on imaging in a patient with suspected nephrolithiasis. We also review the unique imaging and histopathological findings of this entity and discuss why prophylactic surgery is considered the treatment of choice.

## Introduction

Bronchogenic cysts are rare lesions that originate from the primitive foregut during weeks 3–7 of embryonic development.^1^ These lesions are one of the most common malformations of the lower respiratory tract, and hence mostly occur within the thoracic cavity. However, cervical and abdominal cysts have also been seen.^2^ We report the case of a bronchogenic cyst located in the retroperitoneum. Retroperitoneal bronchogenic cysts (RBs) are thought to originate from an anomaly in the budding of the bronchial tree^3^ and initially were discovered in 1953 by Miller et al.^4^ Fortunately, RBs are mostly benign and asymptomatic.^3^ Through this article, we aim to educate our audience about this rare entity and its management.

## Case

An 18-year-old male with a history of recurrent nephrolithiasis presented to the emergency department with severe left back pain radiating to his groin. The patient had experienced similar episodes in the past, but the pain would typically subside shortly following the excretion of a renal stone. Since his current episode persisted beyond 3 h, the patient decided to visit the emergency department. Here, an unenhanced CT incidentally identified a left suprarenal mass. At this point, the patient was referred to our institution for further management.

**Figure 1.**
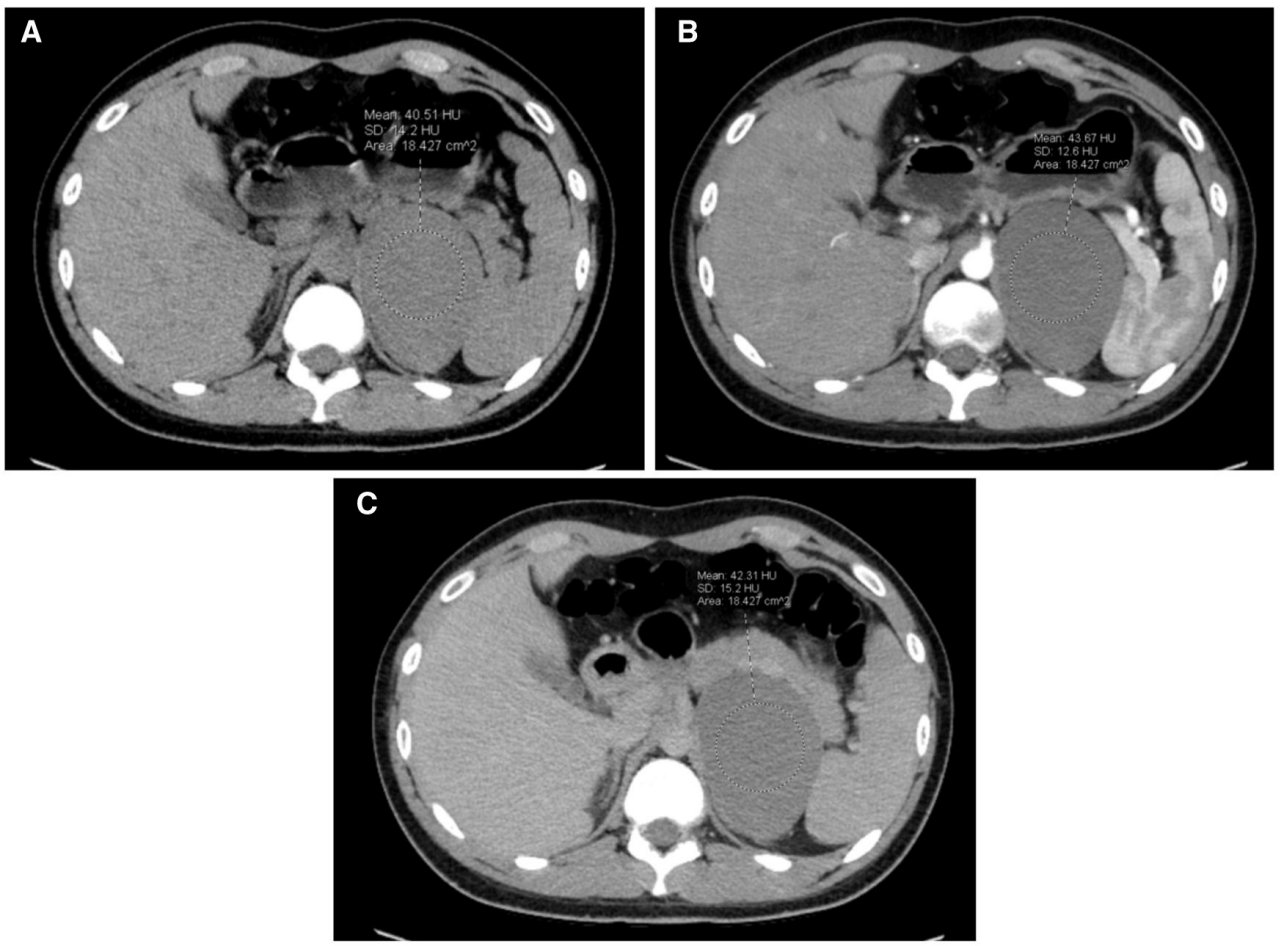
Axial pre-contrast (A), post-contrast late arterial phase (B), and post-contrast delayed phase (C) CT images demonstrating a nonenhancing, homogenous, well-circumscribed, thin-walled cystic mass. The lesion shows a central attenuation of 41 HU in the pre-contrast CT image, 44 HU in the post-contrast late arterial phase CT image, and 43 HU in the post-contrast delayed phase CT image.

**Figure 2.**
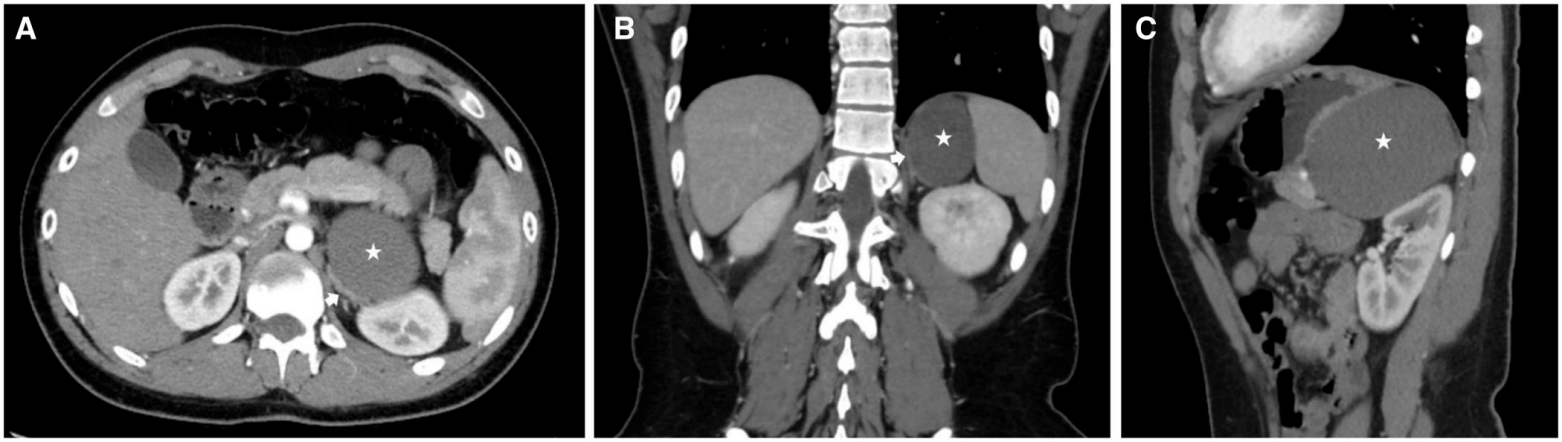
Axial (A), coronal (B), and sagittal (C) post-contrast CT images showing a left retroperitoneal suprarenal cyst (star) pushing the left kidney posteroinferiorly, the left adrenal gland (A, B; white arrow) medially, and the stomach superoanteriorly. The cyst is adjacent to the left adrenal gland, pancreas, and the stomach. There is no fat tissue between them.

The mass was suspected to be an adrenal cyst; however, differential diagnosis also included pseudocyst, gastrointestinal duplication cyst, and cystic adrenal neoplasm. CT with contrast confirmed the lesion to be a 9.6×6.9 cm left suprarenal mass. The lesion was non-enhancing on pre-contrast phase, homogenous, thin walled, well circumscribed, with possible haemorrhagic or proteinaceous change (Figure 1), without mural nodules or septations. The origin of the mass was uncertain, but neighbouring structures included the left adrenal gland, pancreas, spleen, left kidney, gastric wall, and proximal small bowel (Figure 2). Additionally, several non-obstructive renal stones were observed bilaterally in the patient’s non-hydronephrotic kidneys. The pain subsided with the help of analgesics, and the patient was referred to the clinic and subsequently discharged.

The patient returned to the emergency department 5 days later with recurrent abdominal pain. A CT without contrast ruled out possible complications that could have occurred secondary to his nephrolithiasis. The CT identified bilateral punctate renal densities thought to be calculi, and a hyperdense left suprarenal mass with a central attenuation of 42 HU.

Laboratory investigations performed during the first encounter were found to be within normal limits. These included a comprehensive metabolic panel, metanephrine, androgen, cortisol, lactate dehydrogenase, and a dexamethasone suppression test. Therefore, the cyst was postulated to be benign, non-functioning and asymptomatic in nature. Subsequently, the patient underwent a left posterior retroperitoneoscopic cystectomy of the mass. His pain improved and he was discharged on oral hydrocodone-acetaminophen as needed.

Post-surgical pathologic examination demonstrated a soft, round, smooth mass filled with white creamy fluid that was non-adherent to the surrounding structures. Microscopically, smooth muscle and a distorted possible epithelial lining were seen along one edge. Histology results were found to be consistent with RB.

## Discussion

Bronchogenic cysts are thought to result from an abnormality during embryogenesis. They are almost evenly distributed amongst both genders and have been seen in patients ranging from 59 years old to as young as a 25-week-old embryo.^2^ Bronchogenic cysts occur mainly in the mediastinum, although rarely retroperitoneally, as with our patient.^5^ Most of these cysts occur in the left adrenal gland and pancreatic region, typically measuring less than 5 cm in diameter.^6^

Embryologically, bronchopulmonary buds are derived from the primitive foregut during weeks 3–7 of embryogenesis. During weeks 4–5 of gestation, the primitive diaphragm also begins to form, and a multitude of embryonic structures fuse to eventually partition the thoracic cavity from the abdominal cavity, thereby sealing the canals previously connecting the cavities. Notably, the left canal is larger, and hence closes later than the right canal, which is why most RBs, including this case, occur on the left.^5^

RB is mostly an asymptomatic incidental imaging finding, as was the case with our patient.^1^^,^^2^ Cyst secretions may accumulate, producing compressive symptoms like nausea, vomiting, abdominal pain, early satiety, infection, perforation, and haemorrhage. Pheochromocytoma-like symptoms may also be observed due to impingement on the adrenal gland,^1^ especially in cysts measuring over 7 cm.^6^

A diagnostic approach combining clinical, imaging, laboratory, and histopathology findings is crucial to accurately diagnose RB. This is due to its broad differential including benign and malignant tumours, metastasis, cysts, infections, teratomas and bronchopulmonary sequestrations, especially if the cyst is infected, large, or compressing adjacent structures.^1^^,^^7^ Pathologies like adrenal adenoma, pheochromocytoma, peripancreatic cysts, carcinoid tumour, abdominal malignancies may be ruled out via laboratory work. Despite its location, endocrine panels are typically normal, as with our patient.^5^ Notably, CEA is a normal bronchial mucus component which spills into blood, falsely elevating serum carcinoembryonic antigen levels.^8^

The combination of MRI with Multi-Detector CT (MDCT) is considered optimal for diagnosis, although other modalities may also be considered.^1^ Ultrasound (US) is useful in well-transduced calcified lesions, appearing as anechoic masses with possible hyperechoic debris. Unfortunately, US is not preferred due to difficult retroperitoneal access, gastrointestinal gas, and varying internal echogenicity.^2^

On CT, RB presents as either a complete adrenal structure, fusiform, or calcified cyst.^1^ RB appears as a homogenously hypoattenuating, non-enhancing, sharply defined spherical lesion with well-circumscribed smooth or lobulated borders.^1^ RB contains water and proteinaceous mucus with possible remnants of prior haemorrhage or infection, with an attenuation comparable to that of water (0-20 HU). However, hyperattenuation with coefficients of up to 120 HU are possible due to haemorrhage, calcification, or thick proteinaceous mucin,^5^ as was seen in this case. Notably, fluid-fluid levels result from high-viscosity at the cyst bottom.^5^

Unfortunately, CT alone may be inadequate to diagnose lesions lacking internal heterogeneity or mural enhancement. Here, MRI combined with MDCT is diagnostic based on location, neighboring structures, shape, size, wall thickness, calcifications, septa, and fat.^1^ Hyperattenuating cysts may be mistaken for solid masses with variable intermediate-to-high signal intensity on CT and T1-weighted MRI; here, a T2-weighted MRI will display long T2-weighted relaxation times with an extremely high signal intensity.^1^^,^^5^ This is due to paramagnetic entities within RB, including protein and haemorrhagic methaemoglobin, not found in simple cysts. Additionally, these lesions can be distinguished from teratomas as they lack signal intensity inversion on T1-weighted fat-suppressed imaging.^5^

Definitive diagnosis of RB is on histology. Bronchogenic cysts are defined by the simultaneous presence of ciliated respiratory epithelium, mucinous glands, and well-differentiated cartilage.^1^ Various treatment options exist. Prophylactic removal of the retroperitoneal mass prevents subsequent surgical complications including cyst infection; malignant lesion being misdiagnosed as benign; and malignant degeneration of unremoved cysts.^5^ Lesions latching on to neighbouring viscerae may require en bloc resection with possible organ removal,^3^^,^^5^ whereas laparoscopy is preferred by some, due to a small incision size, reduced hospital stay, and lower postoperative costs and complications.^3^^,^^5^^,^^9^ Additional novel techniques include the minimally invasive retroperitoneoscopy, as was performed in our case.^5^ Fortunately, there have been no reports of recurrence.^1^ Rarely, malignant degeneration may be seen; notably, an increased malignancy risk was observed in mal-developed lungs or smokers.^5^^,^^10^

## Conclusion

RB is a rare entity of embryologic origin. Strong clinical suspicion for this cyst should arise if a left-sided retroperitoneal lesion is incidentally found on imaging. MDCT combined with MRI is considered the imaging modality of choice. If the imaging is characteristic of a RB, this should prompt a histological evaluation, since pathology is the gold standard for definitive diagnosis of this lesion. Prophylactic surgical removal is the standard of care, especially considering the malignant potential of the lesion.

## Learning points

- Bronchogenic cyst is a rare entity that originates from the primitive foregut during weeks 3-7 of embryogenesis.
- Rarely, bronchogenic cysts are found below the diaphragm in the retroperitoneum.
- A majority of retroperitoneal bronchogenic cyst occur on the left side.
- MDCT combined with MRI is considered the imaging modality of choice.
- Post-surgical histopathology is the gold standard for diagnosis.
- Retroperitoneal bronchogenic cyst may have potential for malignant transformation.
- Due to the risk for malignant transformation, clinicians should keep retroperitoneal bronchogenic cyst as a part of their differential diagnosis.
- Prophylactic surgical removal is standard of care.
